# Study on the Pharmacological Mechanism of Icariin for the Treatment of Alzheimer’s Disease Based on Network Pharmacology and Molecular Docking Techniques

**DOI:** 10.3390/metabo14010001

**Published:** 2023-12-19

**Authors:** Dongwei Wang, Jilong Zheng, Xingsheng Sun, Liuwei Xie, Yang Yang

**Affiliations:** 1 College of Police Dog Technology, Criminal Investigation Police University of China, Shenyang 110854, China; 2018990116@cipuc.edu.cn (D.W.); 2001990008@cipuc.edu.cn (J.Z.); sunxingsheng@cipuc.edu.cn (X.S.); xieliuwei@cipuc.edu.cn (L.X.); 2 The Second Affiliated Hospital of Shenyang Medical College, Shenyang 110031, China

**Keywords:** icariin, P13K/AKT/MAKP/mTOR signaling pathway, autophagy, Alzheimer’s disease

## Abstract

The purpose of this study is to explore the pharmacological mechanism of icariin (ICA) in the treatment of Alzheimer’s disease (AD) based on network pharmacology and network molecular docking technology. In order to investigate the regulatory effect of ICA on the expression level of AD pathological phosphorylation regulatory proteins, this study further explored the possible molecular mechanism of ICA regulating AD autophagy through network pharmacology. Macromolecular docking network was verified by Autodock Vina 1.1.2 software. The main active ingredients of ICA, the physicochemical properties, and pharmacokinetic information of ICA were predicted using online databases and relevant information. The results showed that the targets of MAPK3, AKT1, HSP90AA1, ESR1, and HSP90AA1 were more critical in the treatment of AD. Autophagy, apoptosis, senescence factors, phosphatidylinositide 3-kinase/protein kinase B (P13K/AKT) signaling pathway, MAKP, mTOR, and other pathways were significantly associated with AD. Docking of ICA with HIF-1, BNIP3, PINK1, and Parkin pathway molecules showed that the key targets of the signaling pathway were more stably bound to ICA, which may provide a better pathway for ICA to regulate autophagy by providing a better pathway. ICA can improve AD, and its mechanism may be related to the P13K/AKT, MAKP, and mTOR signaling pathways, thereby regulating autophagy-related proteins.

## 1. Introduction

Alzheimer’s disease (AD) is a degenerative disease of the central system characterized by progressive cognitive impairment and behavioral injury, which is most frequent in the elderly and preaging. According to research reports, the global prevalence of AD is 3–8% in the elderly aged 65 years, with a higher prevalence in women than in men [1]. AD is primarily characterized by three histopathological features, β-amyloid (Aβ) plaques, hyperphosphorylated Tau protein, and neurofibrillary tangles, along with neuroinflammation mediated by microglial cells [2]. Additionally, observable pathological changes include neuronal atrophy or death, associated astrocytic gliosis, microglial cell activation, and cerebral amyloid angiopathy. These pathological processes ultimately lead to neurodegeneration, resulting in widespread brain atrophy due to synaptic and neuronal loss. AD results in a progressive loss of neuronal function accompanied by an imbalance in brain and body coordination. Its main symptoms include cognitive impairment and memory impairment, which seriously threaten the physical and mental health of patients. AD and dementia have become important social issues and a global priority [3].

Nowadays, drug research for AD in the medical field still faces a challenging situation, and the development of new drugs for AD encounters significant hurdles. Medications targeting cholinergic or glutamatergic neurotransmission are currently available for AD treatment. However, these drugs only provide slight relief for the symptomology, and the efficacy of these drugs remains controversial in clinical trials [4,5]. Newly developed molecular-targeted drugs target Aβ and Tau proteins [6]. Among the approved drugs affecting cholinergic transmission are four acetylcholinesterase (AChE) inhibitors: tacrine, donepezil, rivastigmine, and galantamine [7]. These drugs can improve patients’ cognitive abilities, particularly in cases of mild to moderate severity. For the treatment of moderate to severe AD, the FDA approved memantine in 2003. Memantine acts as an N-methyl-D-aspartate (NMDA) receptor antagonist, reducing the observed excitotoxicity in AD induced by excessive glutamatergic neurotransmission [8]. While showing promise in the development of novel drugs for AD treatment, some have failed to reach the market due to unsuccessful clinical trials. Each drug operates through distinct mechanisms, such as BACE I inhibitors that inhibit the enzyme responsible for converting amyloid precursor protein (APP) into Aβ, RAGE inhibitors that prevent Aβ influx, PPAR-γ agonists restoring insulin sensitivity to reduce Aβ formation, or 5HT6 antagonists modulating neurotransmission to improve cognition. In addition, immunotherapy is being explored for developing anti-Aβ and anti-tau protein medications. Research is also ongoing for alternative strategies targeting misfolded tau proteins and neuronal regeneration [9]. It was reported that there exists a significant lag phase between pathological changes in the brain and the onset of symptoms. Initiating treatment during this lag phase or at the first sign of AD symptoms could greatly benefit patients. Therefore, in 2003, the FDA draft guidelines encouraged the inclusion of patients in the initial stages of the disease in clinical trials. However, another challenge lies in diagnosing/identifying such patients for inclusion in clinical trials [10,11].

**Figure 1:**
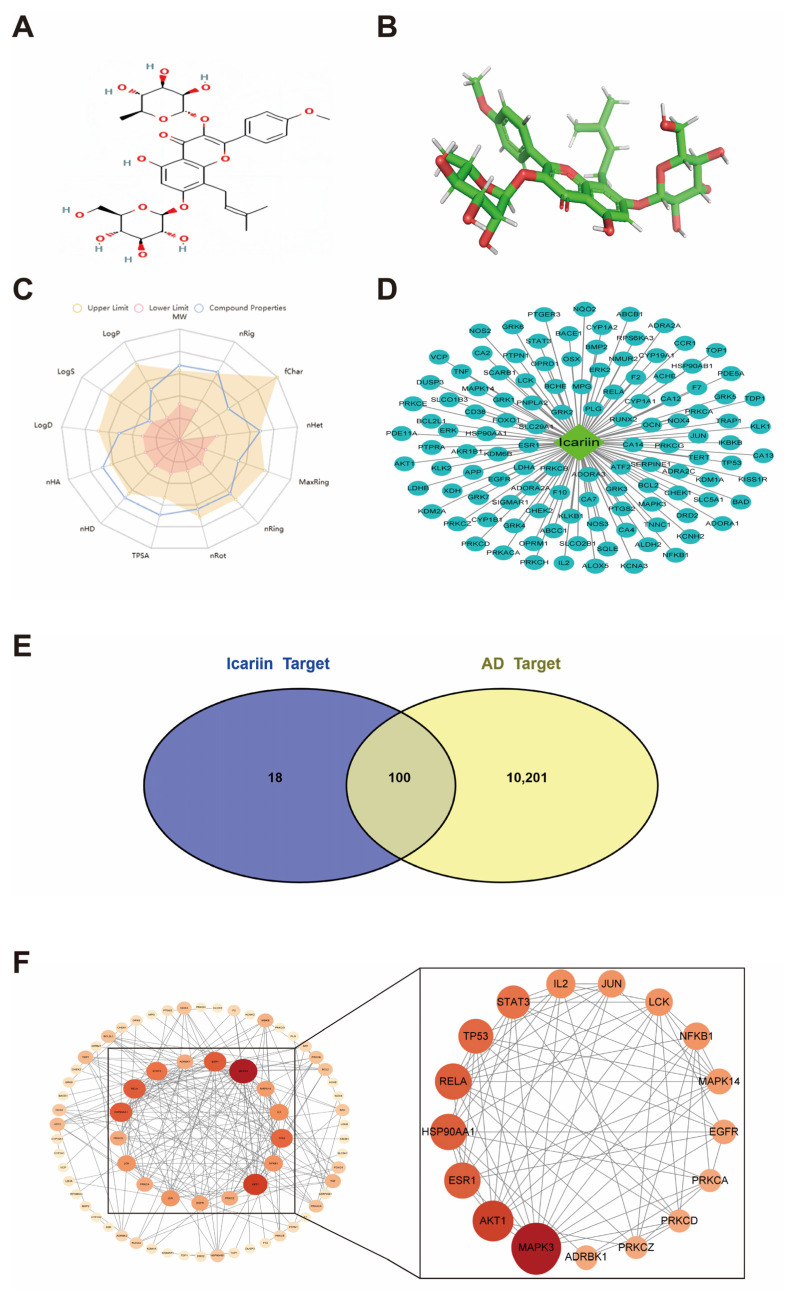
(**A**) is the 2D structure of Icariin. (**B**) is the 3D structure of Icariin. (**C**) is the radar diagram showing the physicochemical properties of Icariin. (**D**) is the “component-target” regulatory network, where light blue oval represents potential targets, while green quadrilateral stands for drug components. There were 118 targets for ICA predicted to build the regulatory network. (**E**) is the intersection of drug targets and AD targets, where yellow represents AD therapeutic targets and blue indicates drug targets. In total, 10,301 disease targets were collected, including 100 intersection targets, suggesting that compound drugs might regulate immune function through these targets. (**F**) is the PPI network. According to the diploid median regulatory targets, the darker color and larger shape indicate that the target was more critical. Core target map is displayed on the right.

Icariin (ICA) (Figure 1A,B) is an extracted component of the natural product Epimedium, which has kidney tonic and anti-aging effects [12,13]. It has various pharmacological effects including osteoclast inhibition, cardiovascular system protection, immune function enhancement, anti-aging, antioxidant, anti-inflammatory, and antibacterial effects [14]. ICA possesses a favorable blood–brain barrier penetrating ability. It exhibits effects on memory enhancement, neuroprotection, prevention of neuronal loss, and improvement in synaptic plasticity to some extent, with the primary site of transformation occurring at the intestinal level [15]. With the continuous deepening of modern research on traditional Chinese medicine, ICA is highly likely to be translated into clinical practice for aging intervention. However, few studies have been conducted in recent years on the pharmacological effects of ICA in delaying aging, particularly in areas highly correlated with age-related cardiovascular and skeletal system aging. Research on the aging of the endocrine system is also limited. In comparison, there is a greater abundance of studies reporting on ICA’s role in neurodegenerative aging, immune senescence, and reproductive aging, though the mechanistic understanding of these areas remains incomplete.

Network pharmacology is a network analysis tool of biological systems to improve the success rate of clinical trials on new drugs and reduce the cost of drug development by selecting specific nodes for multi-target drug molecule design. It emphasizes the multi-pathway regulation of signaling pathways to enhance the therapeutic effect of drugs and reduce their toxic side effects. Network pharmacology, at a systemic level, elucidates the regulatory effects of drugs on the human body’s network. It comprehensively explores the mechanistic actions of drugs on diseases and is currently widely employed to investigate the potential mechanisms underlying the effects of various drugs and their components on multiple diseases [16,17,18]. By adopting molecular docking techniques, the interactions between ligands and receptors can be predicted [19,20]. This paper aimed to screen the core targets of effective ingredients from Epimedium based on network pharmacology, and to adopt a multi-target interaction network and molecular docking techniques to investigate the main active ingredient of Epimedium ICA, identify the signaling pathways related to key targets for the treatment of AD, and explore the potential mechanisms of ICA active ingredients in the treatment of AD.

## 2. Materials and Methods

### 2.1. Prediction of Relevant Targets of AD and Epimedium Components

All protein targets of epimedium components were obtained from the TCMSP (https://old.TCMSP-e.com/TCMSP.php) accessed on 12 May 2022. database. According to the UniProt database, the collected targets were normalized based on their IDs and gene symbols (http://www.uniprot.org/) accessed on 12 May 2022. Moreover, AD-related genes were obtained from Universal card (http://www.Genecard.org/) accessed on 18 May 2022 and OMIM (https://omim.org/) accessed on 19 May 2022 by entering the keyword “Alzheimer’s disease”. Targets were also normalized against the UniProt database. And potential targets for ICA components were acquired through screening the common targets of drugs and diseases (Figure 1D).

### 2.2. Protein–Protein Interaction (PPI) Data

Venny 2.1 was used to obtain the intersection of disease components and targets, which was later imported into STRING (http://stringdb.org/) accessed on 12 May 2022 for protein interaction analysis. The degree values were calculated by the Bisogenet plug-in of Cytoscape 3.8.0 software, and the targets were adjusted according to the diploid median, with a darker color and larger shape indicating a greater importance of targets for constructing the protein–protein interaction networks. Cytoscopy 3.8.0 (http://www.CellLandscape.org/) accessed on 12 May 2022 was employed for analysis to construct the component–target–disease network.

### 2.3. GO and KEGG Pathway Enrichment Analysis

**Figure 2:**
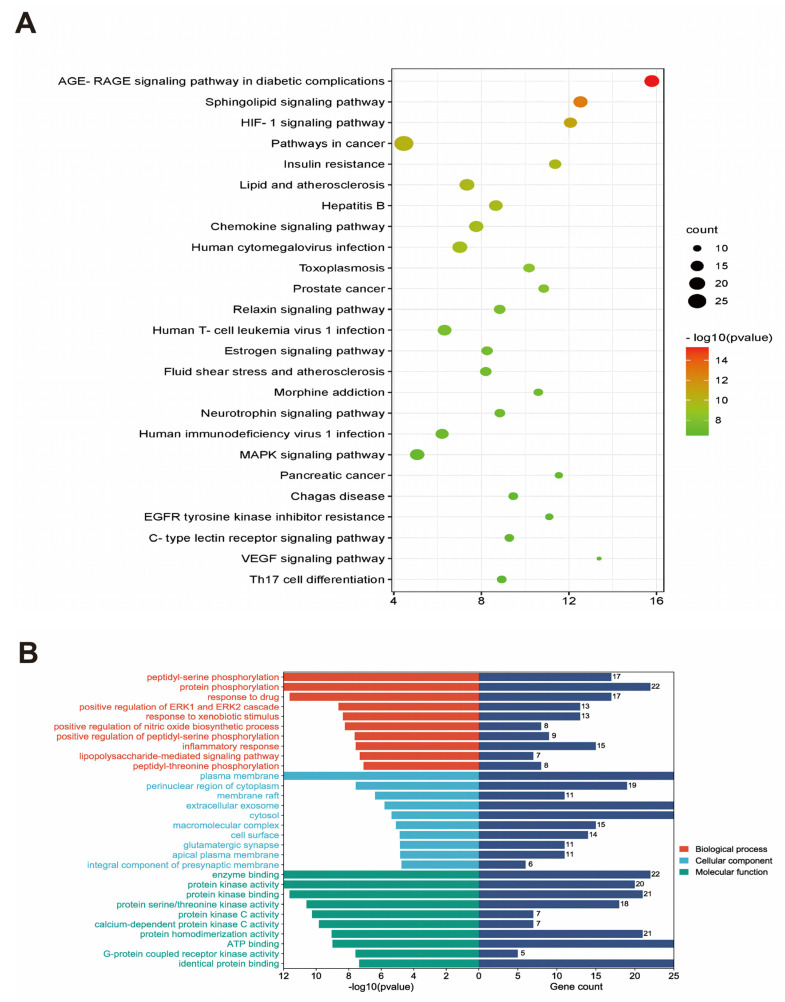
(**A**) shows the result of GO enrichment analysis. (**B**) displays the result of KEGG enrichment analysis.

The DAVID (Database for Annotation, Visualization and Integrated Discovery) database (http://david.abcc.ncifcrf.gov/) accessed on 12 May 2021 is an online, free bioinformatics database used for differential gene function and pathway enrichment analyses. Potential targets obtained based on Gene Ontology Function Annotation (GO) were analyzed online and imported into DAVID, which involved the potential targets and their associated signaling pathways (KEGG). Thereafter, the analysis results were visualized by plotting the bubble plots via the mic-share database (Figure 2A).

### 2.4. Molecular Docking

The PubChem database (https://pubchem.ncbi.nlm.nih.gov/) accessed on 12 May 2021 was loaded with the icariin 2D structure. The PDB database of the receptor protein structure was downloaded. The water of crystallization and other inactive ligands were removed from the receptor protein structure in the PyMOL software (Version 2.5.2). Hydrogen atoms were added and preserved. In this study, the autodock tool 1.5.6 program was loaded, the software was hydrogenated and charged, and the Autodock Vina 1.1.2 software was applied in molecular docking. Since dockingboxsize contains the binding site for the protein, the molecular docking result was selected as the best conformation for docking, which was visualized using PyMOL. Further, the binding energy scores were extracted and ranked.

## 3. Results

### 3.1. Physicochemical Properties and Pharmacokinetic Information of Icariin

ICA was the main chemical component of Epimedium, an 8-prenyl brassinoside analog. Toxicity prediction based on ProTox-II database was reported for the Epimedium glycosides toxicity model, which had an inactive stem cell toxicity and a very active immunotoxicity of 0.98 in organ toxicity. Moreover, it was clearly observed that the nuclear receptor signaling pathways of peroxisome proliferator-activated receptor, estrogen receptor ligand binding domain, estrogen receptor alpha, aromatase, androgen receptor ligand binding domain, the androgen receptor, and aryl hydrocarbon receptor were largely inactive for toxicity. According to the core target information, MAPK3, AKT1, HSP90AA1, ESR1, RELA, TP53, STAT3, IL2, NFKB1, LCK, JUN, MAPK14, EGFR, ADRBK1, PRKCD, PRKCA, and PRKCZ, which ranked high, might be key targets for the treatment of AD.

The radar plot showing ICA physicochemical properties is presented in Figure 1C, while the pharmacokinetic information can be obtained from Table 1 and Table 2 below.

### 3.2. Potential Targets of ICA for AD

Through the “component-target” regulatory network, the light blue oval represents the potential targets of Epimedium. The green quadrilateral represents the active ingredient of Epimedium, and the potential targets of ICA were detected based on STITCH, Swiss TargetPrediction, and Herb database collection prediction. In total, 118 potential targets were detected (Figure 1D). The regulatory network was constructed using Cytoscape 3.8.0 software. Drug targets intersected with AD targets, where the yellow color represents AD therapeutic targets, whereas the blue color stands for drug targets. Altogether, 10,301 disease targets were obtained based on the Genecards and OMIM databases, and were uploaded to the online mapping tool Venn. Finally, 100 common drug–disease targets were obtained from the Venn diagram, suggesting that compound drugs might regulate the immune function via these targets (Figure 1E).

### 3.3. Potential Target PPI Networks

In line with the relationships between ICA and the 100 potentially active targets obtained above, PPI networks were constructed, and the intersecting proteins were uploaded to the string database for analysis. The results were later analyzed using the cytoNCA plugin of Cytoscape 3.8.0 database to calculate the degree value; thereafter, the targets were adjusted according to the twofold median, with a darker color and larger shape indicating higher importance of the target. Afterwards, core targets were screened by a degree greater than the twofold median value (Degree > 9) (Table 3).

Among them, MAPK3, AKT1, HSP90AA1, ESR1, RELA, TP53, STAT3, IL2, NFKB1, LCK, JUN, MAPK14, EGFR, ADRBK1, PRKCD, PRKCA, and PRKCZ had higher degree values, indicating that they might be the key targets for the treatment of AD (Figure 1F).

### 3.4. Results of GO Analysis and KEGG Metabolic Pathway Enrichment Analysis

To explore the molecular mechanisms underlying the effect of ICA on AD, 100 core targets were subjected to GO (Figure 2B) and KEGG pathway analyses. The most significant *p*-values are shown as bubble plots and bar graphs (Figure 2A). According to the KEGG enrichment analysis results, AD was highly correlated with HIF-1 expression and epimedium was mainly associated with the HIF-1 pathway in the pathogenesis of AD. HIF-1 is closely related to MAPK, mTOR, and P13K-Akt signaling pathways. Of them, MAPK, a serine protein kinase, can be activated by signals from cytokines, neurotransmitters, and hormones, and it has effects on regulating gene expression, cell proliferation, and cell death. The enrichment results suggested that protein phosphorylation in response to drugs, plasma membranes, enzyme binding, and molecular functions focusing on protein kinase activity, protein serine, and threonine kinase activity was mainly enriched. Moreover, the significantly enriched signaling pathways were mainly the HIF-1 signaling pathway and neurotrophic factor signaling pathway. These data suggested a potential mechanism for the pooled effect of ICA on the HIF-1 signaling pathway and neurotrophic factor signaling pathway (Figure 2).

### 3.5. Construction of the Compound-Target-Pathway-Disease Pharmacology Network for Compound ICA

**Figure 3:**
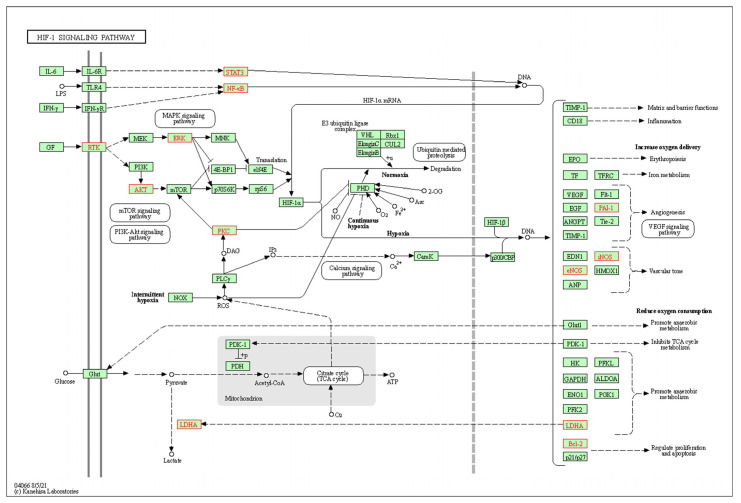
The potential target enrichment in the HIF-1 signaling pathway.

Two Excel data files and one phenotype file were created by using compound ICA, potential AD-related targets, AD-related pathways, and AD as nodes. In addition, the relevant pharmacological networks were constructed and visualized with Cytoscape 3.8.0 software. As a result, ICA acted on AD via 100 targets and involved different signaling pathways, indicating that ICA exhibited multi-target and multi-pathway characteristics. Meanwhile, the targets were mainly enriched in the HIF-1A, BNIP3, PINK1, and Parkin pathways (Figure 3).

### 3.6. Molecular Docking Validation of Core Targets and Active Compounds

**Figure 4:**
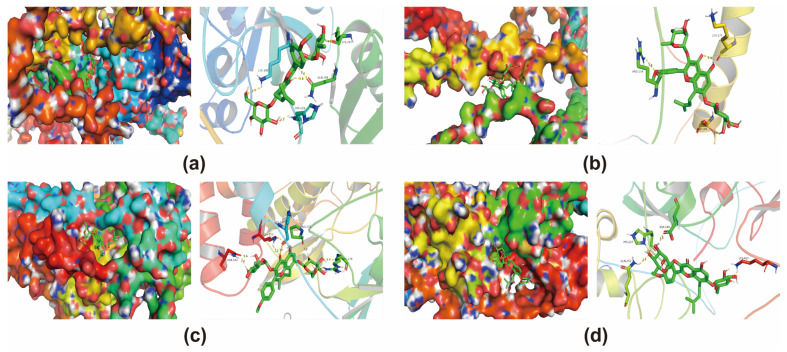
Docking conformation of Icariin with molecules related to the HIF-1A/BNIP3/PINK1/Parkin pathway. (**a**) Docking conformation of HIF-1A with Icariin, (**b**) docking conformation of NIP3 with Icariin, (**c**) docking conformation of PINK1 with Icariin, and (**d**) docking conformation of Parkin with Icariin.

The core targets of ICA against AD were screened by employing the PPI network. To validate the reliability of candidate targets in the PPI network, the AlphaFold predictions collected from the Uniprot database were utilized uniformly for all protein structures, since some of them were not resolved yet. Moreover, docking conformations of ICA with HIF-1, BNIP3, PINK1, and Parkin pathway molecules were performed, respectively (Table 4, Figure 4).

## 4. Discussion

AD is a central neurological disease that can lead to progressive cortical functional degeneration, and it is pathologically characterized by the formation of senile plaques in the brain from extracellular β-like amyloid deposits and intracellular tau protein hyperphosphorylation, eventually leading to intracellular fiber tangles in neuronal cells and a loss of neurons [21]. Continuous progression will result in problems with memory, language, and visuospatial deficits. At present, the main types of drugs used to treat AD are M-type cholinergic receptor agonists, cholinesterase inhibitors, antioxidant drugs, and inhibitors of amyloid deposition. ICA is extremely antioxidative, which can be potentially used in the treatment of AD disorders through inhibiting excessive oxidation, scavenging free radicals, and repairing mitochondrial DNA damage, possibly via the HIF-1A, BNIP3, PINK1, and Parkin pathways. Some studies have shown that ICA possesses cardiovascular protective functions and anti-aging effects [22]. Recent studies have shown the potential of ICA as a natural compound against neurodegenerative diseases; thus, ICA is also involved in pharmacological effects on factors related to the pathophysiology of AD and PD [23].

The Chinese herbal medicine Epimedium contains a variety of chemical components and trace elements such as Epimedium glycosides, Epimedium elements, as well as Epimedium polysaccharides, and it exhibits numerous therapeutic effects. It has been widely used in clinical applications, with multi-target effects and few side effects, typically, and it has demonstrated good prospects in the treatment of complex AD diseases [24]. The pharmacological mechanism of Epimedium in the treatment of AD was explored through an in-depth study on network pharmacology and the construction of a “drug-target-disease” pharmacological network involving multiple targets and pathways. Based on the predictions collected from STITCH, Swiss Target Prediction, and Herb database, a total of 118 potential ICA targets were identified. In the meantime, 10,301 disease targets were acquired from Genecards and OMIM databases, including 100 intersecting drug–disease targets, and key targets were selected in the pivotal PPI network. Based on the molecular docking conformation prediction by known protein structures, the molecular docking conformations of ICA and HIF-1A/BNIP3/PINK1/Parkin pathway were predicted, respectively. As suggested by the molecular docking simulations, ICA, which acted on the HIF-1 signaling pathway, might have potential effects on AD via HIF-1A, BNIP3, PINK1, and Parkin pathways. Additionally, the KEGG enrichment analysis results indicated that AD was highly correlated with HIF-1 expression and that epimedoside was closely associated with the HIF-1 pathway in the pathogenesis of AD [25]. Typically, HIF-1 is closely related to MAPK [26], mTOR [26], and P13K-Akt [27] signaling pathways. MAPK, the serine protein kinase, has an impact on regulating gene expression, cell proliferation, and cell death, and it can be activated via signals from cytokines, neurotransmitters, and hormones [28]. The neuroinflammation caused by AD is mainly manifested as the over-activation of microglia and thus the release of pro-inflammatory factors. Inhibiting the production and release of pro-inflammatory factors can effectively slow down the progression of AD [29]. The MAPK signaling pathway is activated after NF-κB is directly involved in the transcription and translation of pro-inflammatory factors, which can promote the production and release of downstream inflammatory factors, ultimately leading to neuroinflammation. The stimulation of AD triggers the occurrence of neuroinflammation; therefore, inhibiting the MAPK pathway can improve neuroinflammation to some extent, thus reducing neuroinflammation in AD disease [30,31]. Moreover, suppressing the MAPK pathway has the effect of improving the disease model associated with neuroinflammation.

mTOR, also called mammalian rapamycin target protein, is a serine and threonine kinase. mTOR activates the PI3K/Akt signaling pathway and is involved in cell proliferation, differentiation, and apoptosis. It functions to sense the cellular energy availability and regulate cell proliferation [32]. Studies have shown that mTOR can activate autophagy as a negative regulator of autophagy, and its expression level is closely related to the autophagy function of neuronal cells. It has been reported that mTOR signaling is overactive in the brain regions of AD patients, and the phosphorylation status of specific proteins downstream of mTOR can reflect the activity of mTOR. The microglial mTOR signaling pathway is closely related to Trem2 regulation and lysosomal biogenesis, and the up-regulation of microglial Trem2 by mTOR could be a better way to treat β-amyloid-associated AD disease [33]. However, because mTOR is controlled by the upstream PI3K-AKT pathway, an excessive activation of mTOR can lead to Aβ accumulation. On the other hand, the abnormal activation of mTOR, a hub for the regulation of intracellular energy metabolism, can cause enhanced metabolism, like protein synthesis and aerobic glycolysis, in tumor cells. Phosphatidylinositol 3-kinase and protein kinase B are components of numerous signaling pathways, among which, the PI3K-AKT signaling pathway is important in the organism. Studies have reported that the PI3K-AKT signaling pathway is involved in the differentiation, growth, proliferation, and repair of neuronal cells, and is closely associated with AD [34]. It plays a key role in alleviating brain damage in the early stage of stroke, and the inhibition of this pathway can mitigate neuroinflammation in a microglia-mediated inflammatory microenvironment [35]. HIF-1 acts as a “killing factor” and a “protective transcription factor” depending on the severity of hypoxia [36], and it thereby plays a dual role. Initially, HIF-1 is closely related to the regulation of various physiological pathways such as hematopoietic stem cell regulation, cell proliferation, survival, apoptosis, angiogenesis, glucose metabolism, and immune cell activation. Hypoxia-inducible factors can eliminate mitochondrial dysfunction through inducing mitochondrial autophagy and maintaining cellular homeostasis under hypoxic conditions. Severe and prolonged hypoxia will promote the formation and accumulation of Aβ, which can thereby lead to brain damage by inducing cell death and neurodegeneration, resulting in an imbalance of calcium homeostasis in brain neurons and astrocytes, accompanied by neuronal loss or death and microglial activation [37]. Therefore, compounds with hypoxia-inhibiting potential, particularly HIF-1, may be used in the development of treatments for neurodegenerative diseases. Parkin is an E3 ubiquitin ligase, which has been shown to regulate mitochondrial biogenesis and dynamics as well as mitochondria-derived vesicle formation. It is a major signaling pathway regulating autophagy and mediating the clearance of damaged organelles and the maintenance of internal environmental homeostasis. The activation of this signaling pathway enhances mitochondrial autophagy, which is beneficial for the treatment of inflammatory diseases [38]. Parkin gene mutations are suggested to induce the impairment of mitochondrial autophagy, which serves as an important mechanism leading to neurodegenerative pathology such as Parkinson’s disease, AD, and Hunyandon’s disease. Moreover, its mediated mitochondrial autophagy plays an important role in maintaining normal nervous system function and countering damage to the nervous system from peripheral stimuli [39]. BNIP3 is a BH3-only autophagy protein that can induce both cellular autophagy and cell death, and the overexpression of this gene appears to be a key trigger for neuronal death in the CA3 region of the hippocampus [40]. In response to oxidative stress stimulated by aging, the BNIP3 gene regulates the mitochondrial autophagy of neurons in the hippocampal CA3 region, resulting in neurodegeneration, and it plays a key role in activating unique neuronal protection programs or death.

PINK1 is a protein kinase particularly expressed in the whole cells of the body, heart, muscle, and brain, all of which are high-energy-consuming organs [41]. In the identification of the phosphatase and tensin homologue-induced kinase, PINK1 was found to be essential for neuronal survival [42], and PINK1 down-regulation was found to be closely associated with mitochondrial morphological function and neuronal-like differentiation. Furthermore, mitochondria are one of the important energy sources and have a critical effect in the study of AD progression, and PINK1 can attenuate AD via mitochondrial autophagy via A deposition in AD [43,44]. The main features of AD are persistent mitochondrial dysfunction, cognitive decline, and inevitable memory loss. Autophagy is a common phenomenon occurring in the physiological and pathological life activities of human body, which fuses with lysosomes to form autophagic lysosomes that degrade their contained contents in a process involved in aging and AD pathogenesis. It is found that autophagy has become a new target for the treatment of AD, and mice lacking autophagy are prone to protein aggregation and neurodegeneration in neurons [45]. Moreover, impaired autophagy can induce the abnormal accumulation of Aβ, which exacerbates oxidative stress and the inflammatory responses by generating reactive oxygen species and activating microglial cells, eventually exacerbating neuronal dysfunction and leading to the development of pathological neuronal damage [46,47].

The cholinergic system participates in cognitive processes, and the dysfunction of this system is a contributing factor to various dementias, including AD. Cholinergic neurons in the Meynert basal nucleus selectively exhibit deposits of amyloid plaques and NFT, eventually undergoing degeneration due to the initiation of pro-inflammatory events, further worsening cognitive abilities. Cholinergic deficits also alter blood–brain barrier permeability, leading to improper metabolite transport, hindering the clearance of amyloid plaques and worsening the condition [48]. Changes in the calcium ion permeability of nicotinic acetylcholine receptors (nAChRs) can result in impaired synaptic integrity. In the hippocampus and cortical synaptic regions, the binding of Aβ to α7- and α4β2-nAChRs is maximal [49]. The reduced expression of acetylcholine acetyltransferase and increased expression of acetylcholinesterase (AChE) lead to acetylcholine depletion and dementia deterioration. AChE also interacts with Aβ peptides, promoting plaque formation [50]. The degeneration of noradrenergic neurons is also associated with cognitive impairment and neurodegenerative changes. It was reported that noradrenergic receptors densely exist on astrocytes, the activation of which can enhance synaptic plasticity, improving learning and memory [51]. Serotonin is also involved in the pathogenesis of AD. A loss of serotoninergic neurons and lower levels of this neurotransmitter are observed in the brainstems of many AD patients. Serotonergic inputs from the midbrain raphe nucleus regulate cortical plasticity and memory formation, and dysfunction in this pathway leads to memory loss [52]. Glutamate acts on N-methyl-D-aspartate (NMDA) and α-amino-3-hydroxy-5-methyl-4-isoxazolepropionic acid (AMPA) receptors, critical neurotransmitters for maintaining synaptic plasticity. Imbalance in glutamate/glutamine metabolism leads to sustained neuronal depolarization, excitotoxicity, and synaptic damage. Additionally, Aβ induces hypersensitive reactions in NMDA receptors, disrupting the regulatory control of NMDA activity, and causing excitotoxicity [53]. GABA and serotonin are closely associated in the posterior nucleus, a brainstem region with many serotonergic neuron clusters. A report suggests that 5HT6R antagonists enhance serotonin levels through GABAergic neurons, improving cognitive decline. The same molecules can also reduce plaque formation by decreasing γ-secretase activity without affecting β-secretase [54]. The loss of inhibitory control of GABAergic neurons on cholinergic and glutamatergic neurons is linked to synaptic damage in AD patients. Therefore, the complex interplay of various neurotransmitters is crucial in maintaining cognitive integrity. Imbalances in any of these neurotransmitters may further exacerbate AD symptoms.

Aβ, Tau, and genetic risk factors for AD influence dendritic integrity and disease progression [55]. The formation of amyloid plaques begins at post-synapses. Tau phosphorylation is a protective mechanism against toxic amyloid protein deposits. Phosphorylated Tau dissociates from post-synaptic sites, becoming substrates for other kinases, leading to excessive phosphorylation at various sites. Over-phosphorylated Tau gradually spreads from post-synaptic sites to dendrites and cell bodies, finally diffusing from axons to other neurons, causing synaptic dysfunction, dementia, and neurodegenerative changes [56]. Neuroinflammation plays a central role in the pathogenesis of AD. Acute inflammation has a protective role in defending against brain injuries such as the presence of Aβ plaques. However, the continuous activation of microglial cells hinders plaque removal while retaining their ability to release pro-inflammatory cytokines, resulting in an imbalance between pro-inflammatory and anti-inflammatory cytokines. Aβ deposits activate various Toll-like receptors (TLR2, TLR4, and TLR6) and their co-receptors, including CD36, CD14, and CD47 expressed by microglial cells. The immune system can produce pro-inflammatory cytokines of the IL-1β family, including IL-1β and IL-18, activated by caspase-1 or caspase-8 expression. Inflammasomes such as NLR (NOD-like receptor) family or PYHIN (pyrin and HIN domain-containing) contribute to caspase-1 activation. NLRP3 is a major inflammasome sensing aggregated antibodies. These pro-inflammatory cytokines damage dendritic spines and impede the microglial clearance of Aβ. The induction of inducible nitric oxide synthase by neurons and glial cells, upon encountering pro-inflammatory cytokines, enhances nitric oxide (NO) synthesis, increasing peptide aggregation and making it more effective in inhibiting synaptic plasticity [57]. Under the influence of these cytokines, CDK is activated, leading to Tau protein over-phosphorylation and increased formation of Aβ plaques. Due to the imbalance in GABAergic mechanisms in AD, the inhibitory effect of GABA on activated microglial cells is also lost, further promoting the release of pro-inflammatory cytokines [58]. Other cells, such as endothelial cells, oligodendrocytes, and neurons, also contribute to neuroinflammation. Several inflammatory protective molecules exist in neurons, such as fractalkine, complement defense protein CD59, and CD200 and immune molecules produced by brain endothelial cells, such as IL-1β, IL-6, and CCL2, which can counteract Aβ plaques [59].

## 5. Conclusions

In this study, network pharmacology was used to predict the relevant action targets of ICA in AD. The molecular docking results revealed that ICA exhibited binding activity to the corresponding targets of AD, suggesting its potential to act on AD-related targets and exert therapeutic effects through the HIF-1, MAPK, mTOR, and P13K-Akt pathways. The multi-component, multi-target, and multi-pathway mechanism of action demonstrated by ICA, the primary active component of the natural product Epimedium, serves as a valuable reference for its future application in pharmacological studies. However, as network pharmacology relies on data analysis for prediction, further comprehensive animal and cellular experiments are essential to validate the precise mechanism of ICA in treating AD.

## Figures and Tables

**Table 1:** Physicochemical properties and pharmacokinetic information of Icariin.

Physicochemical Property		Medicinal Chemistry		Absorption		Metabolism	
Molecular Weight (MW)	676.240	QED	0.140	Caco-2 Permeability	−6.178	CYP1A2 inhibitor	---
Volume	644.667	SAscore	4.912	MDCK Permeability	7.7 × 10^−5^	CYP1A2 substrate	---
Density	1.049	Fsp3	0.485	Pgp-inhibitor	---	CYP2C19 inhibitor	---
nHA	15	MCE-18	122.694	Pgp-substrate	+++	CYP2C19 substrate	--
nHD	8	NPscore	1.959	HIA	++	CYP2C9 inhibitor	---
nRot	9	Lipinski Rule	Rejected	F20%	---	CYP2C9 substrate	+
nRing	5	Pfizer Rule	Accepted	F30%	+++	CYP2D6 inhibitor	---
MaxRing	10	GSK Rule	Rejected			CYP2D6 substrate	--
nHet	15	Golden Triangle	Rejected			CYP3A4 inhibitor	---
fChar	0	PAINS	0 alert(s)			CYP3A4 substrate	---
nRig	31	ALARM NMR Rule	2 alert(s)	Distribution		Excretion	
Flexibility	0.290	BMS Rule	1 alert(s)	PPB	64.816%	CL	1.757
Stereo Centers	10	Chelator Rule	0 alert(s)	VD	0.772	T1/2	0.109
TPSA	238.200			BBB Penetration	--		
logS	−3.781			Fu	24.984%		
logP	1.587						
logD	2.066						

Note：For the classification endpoints, the prediction probability values are transformed into six symbols: 0–0.1 (---), 0.1–0.3 (--), 0.3–0.5 (-), 0.5–0.7 (+), 0.7–0.9 (++), 0.9–1.0 (+++).

**Table 2:** Toxicity prediction.

Classification	Target	Shorthand	Prediction	Probability
Organ toxicity	Hepatotoxicity	dili	Inactive	0.74
Toxicity end points	Carcinogenicity	carcino	Inactive	0.83
Toxicity end points	Immunotoxicity	immuno	Active	0.98
Toxicity end points	Mutagenicity	mutagen	Inactive	0.70
Toxicity end points	Cytotoxicity	cyto	Inactive	0.61
Tox21-Nuclear receptor signaling pathways	Aryl hydrocarbon Receptor (AhR)	nr_ahr	Inactive	0.73
Tox21-Nuclear receptor signaling pathways	Androgen Receptor (AR)	nr_ar	Inactive	0.97
Tox21-Nuclear receptor signaling pathways	Androgen Receptor Ligand Binding Domain (AR-LBD)	nr_ar_lbd	Inactive	0.97
Tox21-Nuclear receptor signaling pathways	Aromatase	nr_aromatase	Inactive	0.85
Tox21-Nuclear receptor signaling pathways	Estrogen Receptor Alpha (ER)	nr_er	Inactive	0.88
Tox21-Nuclear receptor signaling pathways	Estrogen Receptor Ligand Binding Domain (ER-LBD)	nr_er_lbd	Inactive	0.99
Tox21-Nuclear receptor signaling pathways	Peroxisome Proliferator Activated Receptor Gamma (PPAR-Gamma)	nr_ppar_gamma	Inactive	0.95
Tox21-Stress response pathways	Nuclear factor (erythroid-derived 2)-like 2/antioxidant responsive element (nrf2/ARE)	sr_are	Inactive	0.89
Tox21-Stress response pathways	Heat shock factor response element (HSE)	sr_hse	Inactive	0.89
Tox21-Stress response pathways	Mitochondrial Membrane Potential (MMP)	sr_mmp	Inactive	0.84
Tox21-Stress response pathways	Phosphoprotein (Tumor Suppressor) p53	sr_p53	Inactive	0.73
Tox21-Stress response pathways	ATPase family AAA domain-containing protein 5 (ATAD5)	sr_atad5	Inactive	0.97

**Table 3:** Core target information.

Gene Symbol	Degree	Betweenness	Closeness	Eigenvector	Information	LAC	Network
MAPK3	28	1213.18	0.26	0.34	3.35	6.71	18.45
AKT1	22	371.72	0.25	0.29	3.26	6.36	14.02
HSP90AA1	19	810.08	0.25	0.23	3.19	4.53	8.41
ESR1	19	557.74	0.25	0.24	3.19	5.68	11.20
RELA	19	398.36	0.25	0.26	3.19	6.63	12.23
TP53	18	776.05	0.25	0.21	3.16	3.89	8.48
STAT3	17	229.70	0.24	0.25	3.14	6.35	9.86
IL2	14	63.35	0.24	0.22	3.03	6.29	8.73
NFKB1	13	165.17	0.23	0.18	2.99	4.92	6.70
LCK	13	48.24	0.24	0.21	2.99	6.31	8.17
JUN	13	309.10	0.24	0.20	2.99	6.00	7.71
MAPK14	12	33.53	0.23	0.22	2.94	7.17	8.25
EGFR	11	252.93	0.24	0.15	2.89	3.45	4.26
ADRBK1	10	923.55	0.23	0.05	2.83	1.00	4.22
PRKCD	10	248.02	0.24	0.15	2.83	3.00	3.42
PRKCA	10	202.93	0.24	0.14	2.83	3.60	4.21
PRKCZ	10	13.82	0.22	0.15	2.83	5.00	5.92

**Table 4:** Molecular docking results.

	HIF-1	BNIP3	PINK1	Parkin
Icariin	−8.0	−7.6	−8.1	−8.2

## Data Availability

The data presented in this study are openly available in TCMSP and UniProt database at https://old.TCMSP-e.com/TCMSP.php and http://www.uniprot.org/ (accessed on 12 May 2021).
